# Persistent luminescence phosphor as *in-vivo* light source for tumoral cyanobacterial photosynthetic oxygenation and photodynamic therapy

**DOI:** 10.1016/j.bioactmat.2021.08.030

**Published:** 2021-09-04

**Authors:** Meiqi Chang, Wei Feng, Li Ding, Hongguang Zhang, Caihong Dong, Yu Chen, Jianlin Shi

**Affiliations:** a State Key Laboratory of High Performance Ceramics and Superfine Microstructure, Shanghai Institute of Ceramics, Chinese Academy of Sciences, Shanghai, 200050, PR China; b Materdicine Lab, School of Life Sciences, Shanghai University, Shanghai, 200444, PR China; c College of Pharmacy, Qiqihar Medical University, Qiqihar, 161006, PR China; d Department of Ultrasound, Zhongshan Hospital, Fudan University, and Shanghai Institute of Medical Imaging, Shanghai, 200032, PR China

**Keywords:** Photodynamic therapy, Cyanobacteria, Persistent luminescence, Oxygenation, Irradiation-free

## Abstract

Tumor oxygenation level has been regarded as an attractive target to elevate the efficiency of photodynamic therapy (PDT). Cyanobacterial photosynthesis-mediated reversal of tumor hypoxia could enable an oxygen-boosted PDT, but is limited by scant penetration depth and efficiency of external light. Herein, aiming at the dual purposes of reducing biological toxicity induced by long-term light irradiation and alleviating hypoxia, we here introduce a novel-designed CaAl_2_O_4_:Eu,Nd blue persistent luminescence material (PLM) as the *in vivo* light source after pre-excited *in vitro*. The ingenious construction of blue-emitting PLM with “optical battery” characteristics activates cyanobacterial cells and verteporfin simultaneously, which performs the successive oxygen supply and singlet oxygen generation without the long-term external excitation, resulting in the modulated tumor hypoxic microenvironment and enhanced photodynamic tumor proliferation inhibition efficiency. Both *in vitro* cellular assessment and *in vivo* tumor evaluation results affirm the advantages of self-produced oxygen PDT system and evidence the notable antineoplastic outcome. This work develops an irradiation-free photosynthetic bacteria-based PDT platform for the optimization of both oxygen production capacity and light utilization efficiency in cancer treatment, which is expected to promote the clinical progress of microbial-based photonic therapy.

## Highlights

• Construction of CaAl_2_O_4_:Eu,Nd PLM to generate ^1^O_2_ without the aid of exogenous light excitation.
• Cyanobacteria with light-triggered oxygenation effect were employed for the normalization of tumor microenvironment.
• A distinct exogenous “irradiation-free” cyanobacteria-based PDT platform was rationally engineered.

## Graphical abstract

A distinct exogenous “irradiation-free” cyanobacteria-based PDT platform is rationally engineered for ameliorating the tumor hypoxic microenvironment and achieving the successive singlet oxygen (^1^O_2_) generation without the need of exogenous light excitation, which provides a specific paradigm of microbial-based nanotherapy with the assistance of rational design, engineering and integration of persistent luminescence phosphors as the desirable light irradiation source.

**Figure:**
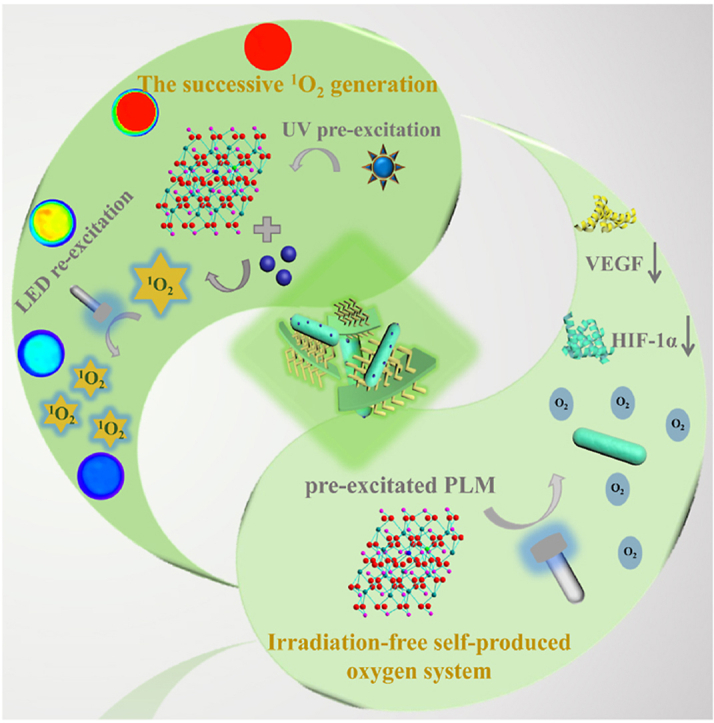

## 1 Introduction

As a typical hallmark of solid tumors, hypoxia caused by abnormal tumor vasculature would induce adaptive metabolic reprogramming and regulate cellular redox state [1,2]. The imbalance of oxygen supply and consumption caused by hypoxia and the produced metabolites from anaerobic glycolysis process give rise to cell proliferation and metastasis, thereby, decreasing the oxygen-reliable and reactive oxygen species (ROS)-dependent therapeutic efficacy [[3], [4], [5]]. Particularly, a series of research data have confirmed that the downregulated intra-tumoral oxygen pressure and severe hypoxic microenvironment contribute to the poor prognosis of type-II photodynamic therapy (PDT) [6,7]. To date, one of the developed and available oxygen production strategies dominantly focuses on the catalytic chemical reaction of nanomaterials (MnO_2_ [[8], [9], [10]], MnO [11], Fe-based catalysts [12,13], Pt nanozyme [14,15], Cu-based materials [16], *etc.*) with endogenous tumor-overexpressed H_2_O_2_. However, the limited intra-tumoral H_2_O_2_ content (10^−4^-10^−3^ M) is still a stumbling block on the path of clinical translation [17,18].

The rapid development of microbial nanomedicine has speeded up the biomedical applications of functionalized microorganisms [[19], [20], [21]]. In 2017, Woo et al. innovatively utilized the photosynthetic and photoautotrophy characteristics of cyanobacterium (*Synechococcus elongatus*) to achieve the maintenance of myocardial metabolism and the improvement of cardiac function, opening a channel for the medical utilization of photosynthetic bacteria [22]. Recently, light-triggered biosynthesis as a prospective oxygen-generating modality has been employed for the normalization the tumor microenvironment [23]. As a victorious paradigm, engineering construction of photosynthetic bacteria, such as red blood cell membrane-algae [24], Ce6-cyanobacterial cells [25], indocyanine green-cyanobacteria biomimetic system [26], and Chlorella-based oxygen-affording engines [27] exhibits the intriguing PDT performance due to adequate supply of exogenous oxygen and inherent tumor-targeting property of bacteria.

As an important trigger element in photosynthetic bacteria-based PDT, light source undertakes the dual tasks of photosynthesis and photosensitizer excitation. Chlorophyll endows cyanobacteria with a broad visible light absorption response for photosynthetic oxygen production [28]. Most of the available researches concentrate on the red-emission (660 nm laser)-assisted cyanobacteria oxygenation and excitation of photosensitizer considering the critical issue of low tissue penetration depth of visible light. Alternatively, upconversion materials are selected as an effective light conversion matrix to further achieve the improved tumor penetration by near infrared (NIR) activation [29]. However, the overheating effect caused by long-lasting laser irradiation would cause non-negligible risk for *in vivo* application [30]. In accompany with the existence of undesirable penetrating depth of NIR for treating deep-seated tumor. Therefore, the optimal design of the light source and its adaptation to the response wavelength of cyanobacteria and photosensitizers are still the current enormous challenge. In addition, the available research on persistent luminescence materials used in the biological field are mainly concentrated in the ZnGa_2_O_4_-based red emission system [31,32]. However, the relatively short persistent luminescence time arising from small particle sizes hinders its further anti-cancer applications. Furthermore, long afterglow materials with other luminous color gamuts are rarely explored.

**Scheme 1:**
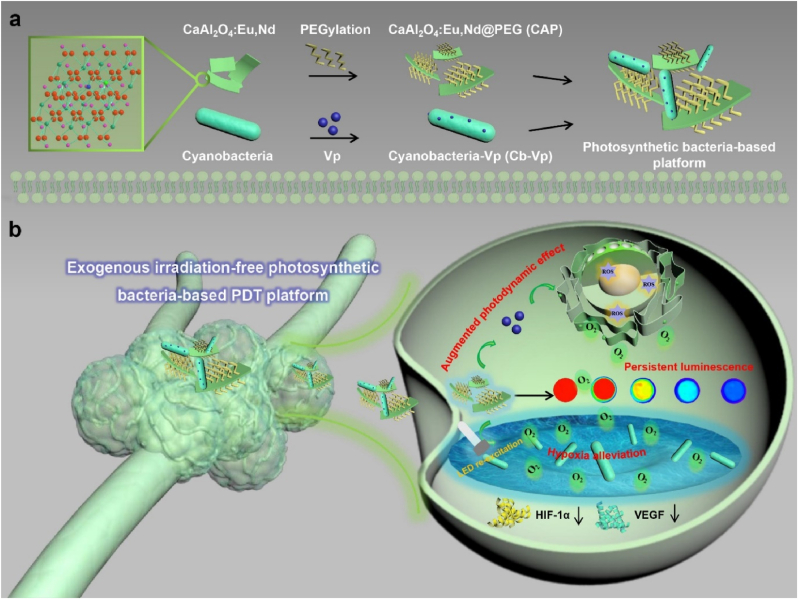
**Experimental design.** Schematic illustration of the co-introduction of CAP and Cb-Vp as the exogenous irradiation-free photosynthetic bacteria-based platform for photodynamic cancer therapy with the continuous output of O_2_ (tumor hypoxia alleviation) and ^1^O_2_ (tumor therapy), achieving the enhanced photodynamic therapeutic effects and outcome.

In this work, a distinct “exogenous irradiation-free” cyanobacteria-based PDT platform was rationally constructed, which could ameliorate the tumor hypoxic microenvironment and achieve the successive singlet oxygen (^1^O_2_) generation without the need of exogenous light excitation through the introduction of CaAl_2_O_4_:Eu,Nd persistent luminescence material (CAO PLM) (Scheme 1). Under the pre-excitation of UV lamp and subsequent LED re-excitation process, CAO PLM exhibits a broad blue persistent emission arising from the 5d-4f electron transition of Eu^2+^ and the existence of electron traps. The energy storage characteristics of PLM and donor emission-acceptor absorption overlap make it possible to simultaneously achieve long-lasting excitation of cyanobacteria and verteporfin photosensitizer, decreasing the expressions of hypoxia-associated HIF-1α and VEGF proteins, relieving tumor hypoxia and potentiating the photodynamic antineoplastic effects. This exogenous irradiation-free platform with “optical battery” characteristic transforms the enduring light-dependent photodynamic treatment mode, providing a constructive ideology for microbial-based photonic tumor therapy.

## 2 Material and methods

### 2.1 Chemicals

CaCO_3_ (99.9%), Al_2_O_3_ (99.9%), Eu_2_O_3_ (99.99%), Nd_2_O_3_ (99.99%), 1-Ethyl-3-(3-dimethylaminopropyl) carbodiimide (EDC) and N-hydroxysuccinimide (NHS) were purchased from Aladdin (Shanghai, China). 1,3-Diphenylisobenzofuran (DPBF), NaOH, 3-aminopropyltriethoxysilane (APTES), N, N-dimethylformamide (DMF), Dimethyl sulfoxide (DMSO) and absolute ethanol were obtained from Sigma-Aldrich. Verteporfin (abbreviation: Vp) was bought from Macklin Inc. 2,7-dichlorofluorescein diacetate (DCFH-DA) was purchased from Medchemexpress. Phosphate buffered saline (PBS), RPMI 1640 medium, penicillin/streptomycin and fetal bovine serum (FBS) were obtained from Gibco, USA.

### 2.2 Synthesis of Ca_0.98_Al_2_O_4_:0.01Eu^2+^, 0.01Nd^3+^ persistent luminescence materials (CAO PLM)

Typically, CAO PLM was synthesized with the high temperature solid state reaction method. CaCO_3_ (9.807 g), Al_2_O_3_ (10.196 g), Eu_2_O_3_ (0.176 g) and Nd_2_O_3_ (0.168 g) were weighed and homogeneously mixed, then ground evenly with a certain amount of ethanol for 30 min and calcined at 1300 °C for 4 h in a reducing atmosphere (5% H_2_ + 95% N_2_).

### 2.3 CAO PEGylation (CAP)

CAO (0.1 g) was dispersed in NaOH (5 mM) solution for overnight stirring, and centrifuged at 7000 rpm for 5 min. Then, the above CAO-OH was re-dispersed into DMF (40 mL) by sonication, and APTES (2 mL) was added under vigorous stirring at 80 °C for 24 h. The products (CAO-NH_2_) were centrifuged, washed with DMF and absolute ethanol several times. Subsequently, EDC (150 mg), NHS (450 mg) and MeO-PEG-COOH (50 mg) were added into PBS buffer (10 mL, 10 mmol L^−1^, pH = 6.0) under vigorous stirring for 2 h. Finally, the above CAO-NH_2_ was re-dispersed and stirred for 12 h. The resulting products were washed several times with water and ethanol.

### 2.4 Cyanobacteria and BG11 culture medium

Cyanobacteria (*Synechococcus* 7942) were obtained from the Freshwater Algae Culture Collection. The stock solutions were prepared as follows: Stock 1: C_6_H_8_O_7_·H_2_O (6.567 g L^−1^), C_6_H_11_FeNO_7_ (6 g L^−1^), EDTANa_2_·2H_2_O (1.107 g L^−1^). Stock 2: H_3_BO_3_ (2.86 g L^−1^), MnSO_4_·H_2_O (1.545 g L^−1^), ZnSO_4_·7H_2_O (0.222 g L^−1^), CuSO_4_·5H_2_O (0.079 g L^−1^), Na_2_MoO_4_·2H_2_O (0.391 g L^−1^), CoCl_2_·6H_2_O (0.0404 g L^−1^). Stock 3: K_2_HPO_4_·3H_2_O (52 g L^−1^), Na_2_CO_3_ (20 g L^−1^). Then, cyanobacteria were cultured in sterile 1 × BG11 medium containing NaNO_3_ (1.5 g), MgSO_4_·7H_2_O (0.075 g), CaCl_2_·2H_2_O (0.036 g), HEPES (4.76 g), Stock 1 (1 mL), Stock 2 (1 mL), Stock 3 (1 mL) at 25 °C under light with oscillation process.

### 2.5 Synthesis of cyanobacteria-Vp (Cb-Vp)

Vp (2 mg) was mixed with EDC aqueous solution (6 mL, 1 mg mL^−1^) for 1 h and centrifugated. Then, PBS buffer (15 mL, pH = 7.4) containing cyanobacteria (5 × 10^7^ cell mL^−1^) was added and stirred for 4 h. The resulting product was dialysized (MWCO: 1000 Da) against deionized water for 2 h.

### 2.6 Singlet oxygen (^1^O_2_) detection

DPBF/DMF solution (60 μL, 1 mg mL^−1^) was added into Vp/DMF solution (3 mL, 1 μg mL^−1^). Then, CAP aqueous solution (100 μL, 30 mg mL^−1^) was added into the above solution after UV pre-irradiation for 10 min. The absorption spectra of DPBF were measured.

### 2.7 ESR measurement

The ESR experiments were performed with the assistance of the trapping agent 2,2,6,6-tetramethyl-4-piperidone hydrochloride (TEMP, Dojindo). The mixtures of CAP, Cb-Vp and diluted TEMP (200 μM) were irradiated with white LED lamp (1360 lm) for different irradiation durations, then tested by an electron paramagnetic spin spectrometer.

### 2.8 Cell culture

4T1 cancer cells were purchased from the Cell Bank of Shanghai Institute of Biochemistry and Cell Biology, Chinese Academy of Sciences. 4T1 cancer cells were cultured in RPMI 1640 medium supplemented with 10% fetal bovine serum and 1% penicillin/streptomycin at 37 °C in a humidified atmosphere with 5% CO_2_.

### 2.9 *In vitro* cytotoxicity

4T1 cancer cells were seeded into a 96-well plate and incubated at 5% CO_2_ and 37 °C for 24 h. Fresh cell culture mediums respectively including Vp (0.06 μg mL^−1^), Cb-Vp (Vp: 0.06 μg mL^−1^, Cb: 2 × 10^7^ cell mL^−1^) or Pre-CAP (400 μg mL^−1^) + Cb-Vp (Vp: 0.06 μg mL^−1^, Cb: 2 × 10^7^ cell mL^−1^) were added. After 12 h of incubation, the cells were irradiated with white LED light for different durations. After another 12 h co-incubation, the culture medium was replaced by 100 μL fresh medium containing 10% CCK-8 and cultured for an another 2 h. The absorbance at the wavelength of 450 nm was measured by microplate reader.

### 2.10 Western blot analysis

4T1 cancer cells were seeded into 6-well plates and cultured for 24 h with up to 70–80% confluence. Then the treated 4T1 cancer cells (Pre-CAP [400 μg mL^−1^]; Cb-Vp [Cb:5 × 10^7^ cell mL^−1^, Vp:0.3 μg mL^−1^]; L refers to white LED re-excitation for 2 min) were cultured for 24 h, washed with PBS and lysed to collect the protein. The PVDF membranes were blocked with 5% nonfat dry milk at room temperature for 1 h in the decoloring shaker and then incubated with anti–HIF–1α and anti-VEGF overnight at 4 °C. The membranes were washed for three times in the decoloring shaker. Subsequently, the membranes were incubated with secondary antibodies for 30 min and washed for three times in the decoloring shaker subsequently. ECL reagent was then added and reacted for 1–2 min. The membranes were exposed in a darkroom. The protein expressions were quantified by software Image J.

### 2.11 *In vitro* oxygenation detection

4T1 cancer cells were seeded in a confocal glass bottom-dish (cell density = 10^5^/disk) and cultured for 12 h. Then, they were cultured in hypoxic environment for another 12 h. The cell culture medium was then replaced with fresh culture medium containing Ru(dpp)_3_Cl_2_ probe (10 μg mL^−1^). After incubation at 37 °C for 4 h, the culture medium was replaced with fresh culture medium containing Pre-CAP (400 μg mL^−1^), Pre-CAP (400 μg mL^−1^) + Cb-Vp (Cb: 5 × 10^7^ cell mL^−1^, Vp: 0.3 μg mL^−1^) and cultured in the dark for 2 h. After LED light irradiation (light irradiation groups), the cells were washed twice with PBS. The fluorescence imaging was analyzed through a confocal laser scanning microscope.

### 2.12 Detection of intracellular ^1^O_2_ production

4T1 cancer cells were seeded in a confocal glass bottom-dish (cell density = 10^5^/disk) and cultured. Then, the cell culture medium was replaced with fresh culture medium containing Pre-CAP (400 μg mL^−1^), Cb (5 × 10^7^ cell mL^−1^), Pre-CAP (400 μg mL^−1^) + Cb-Vp (Cb: 5 × 10^7^ cell mL^−1^, Vp: 0.3 μg mL^−1^) and incubated for 12 h. Cells were rinsed carefully by PBS, and then DCFH (10 μM) was added followed by additional incubation in the dark for 40 min. After LED light irradiation (light irradiation groups), cells were washed twice with PBS. The fluorescence imaging was analyzed through a confocal laser scanning microscope.

### 2.13 Detection of live/dead cells

4T1 cells were seeded in a confocal glass bottom-dish (cell density = 10^5^/disk) and cultured. Then, the cell culture medium was replaced with fresh culture medium containing Pre-CAP (400 μg mL^−1^), Cb (5 × 10^7^ cell mL^−1^), Pre-CAP (400 μg mL^−1^) + Cb-Vp (Cb: 5 × 10^7^ cell mL^−1^, Vp: 0.3 μg mL^−1^) and incubated for 12 h. After different treatments (with or without LED irradiation) and cultured for 4 h, the cells were co-incubated by Calcein AM and PI for 1 h, and then imaged using a confocal laser scanning microscope.

### 2.14 Flow cytometry

4T1 cells were seeded in 6-well plates and cultured for 24 h with up to 70–80% confluence. Then, the cell culture medium was replaced with fresh culture medium containing Pre-CAP (400 μg mL^−1^), Cb (5 × 10^7^ cell mL^−1^), Pre-CAP (400 μg mL^−1^) + Cb-Vp (Cb: 5 × 10^7^ cell mL^−1^, Vp: 0.3 μg mL^−1^) and incubated for 12 h. After different treatments (with or without LED irradiation) and cultured for 4 h, treated cells were double stained with Annexin V-FITC Apoptosis Detection Kit followed by the flow cytometer test and analysis.

### 2.15 Animal experiments

4-week-old Female Balb/c mice were purchased from Shanghai SLAC Laboratory Animal Co., Ltd. The Animal experiment were conducted with the approval of ethics by Ethic Committee of Shanghai University. The animal models were established through subcutaneously injecting of 1 × 10^6^ 4T1 cancer cells (100 μL) into the right flank of mouse.

### 2.16 *In vivo* PDT

When the tumor size reached ~100 mm^3^, the mice were divided randomly into five groups (n = 5 in each group): 1. Control (PBS only), 2. CAP + L (10 min UV pre-irradiated CAP (5 mg mL^−1^, 50 μL) and 10 min white LED re-irradiation), 3. Cb + L (Cb (5 × 10^7^ cell mL^−1^, 50 μL) and 10 min white LED irradiation), 4. Cb-Vp + L (Cb-Vp (Cb: 5 × 10^7^ cell mL^−1^, Vp: 0.5 mg mL^−1^, 50 μL) and 10 min white LED irradiation), 5. CAP + Cb-Vp + L (10 min UV pre-irradiated CAP (5 mg mL^−1^, 50 μL) + Cb-Vp (Cb: 5 × 10^7^ cell mL^−1^, Vp: 0.5 mg mL^−1^, 50 μL) + 10 min white LED re-irradiation). The mice were intratumorally injected and treated with different treatment protocols, respectively. On day 2, 4, 6, 8 and 10, Cb cells were replenished and white LED re-irradiation operation was executed. The body weights and tumor sizes were measured every other day. The tumor volume was calculated using the following formula: tumor volume = length × width^2^/2. The mice were sacrificed after two weeks post-treatment and the tumors were collected. Finally, tumors and major organs (heart, liver, spleen, lung, kidney) of different groups were collected and sectioned for H&E staining, TUNEL assay and immunofluorescence labeling (HIF-α, VEGF and Ki-67).

### 2.17 Characterization

SEM images were obtained through scanning electron microscopy (Hitachi, Model S-4800), coupled with an EDX system. TEM images were acquired on JEM-2100F transmission electron microscope. The high-angle annular dark field (HAADF) and annular bright field (ABF) STEM images and the corresponding element mapping were performed on a JEM-ARM 300F Grand ARM (JEOL Company Ltd., Japan). XPS measurement was performed on ESCAlab250 (Thermal Scientific). The crystalline structure of materials was recorded by XRD (Rigaku D/max-B II), using Cu Kα radiation (λ = 0.15405 nm). Fluorescence spectrophotometer (Jobin Yvon, Model FluoroMax-4) was used to collect the persistent luminescence spectra. The UV–vis absorbance spectra were recorded with through UV-3600 spectrophotometer (Shimadzu). The persistent luminescence images were obtained through an IVIS Lumina system. Cell imaging was performed with an FV1000 confocal laser scanning microscope (Olympus, Japan). The change of dissolved oxygen level is monitored by a dissolved oxygen meter (INESA Scientific Instrument, Shanghai).

### 2.18 Statistical analysis

Statistical analysis was conducted through Graphpad Prism 7.00. n.s. represented not significance; *p < 0.05 represented statistical significance; **p < 0.01 represented moderate statistical significance and ***p < 0.001 represented highly statistical significance.

## 3 Results and discussion

### 3.1 Design, preparation and characterization of CAO

**Fig. 1:**
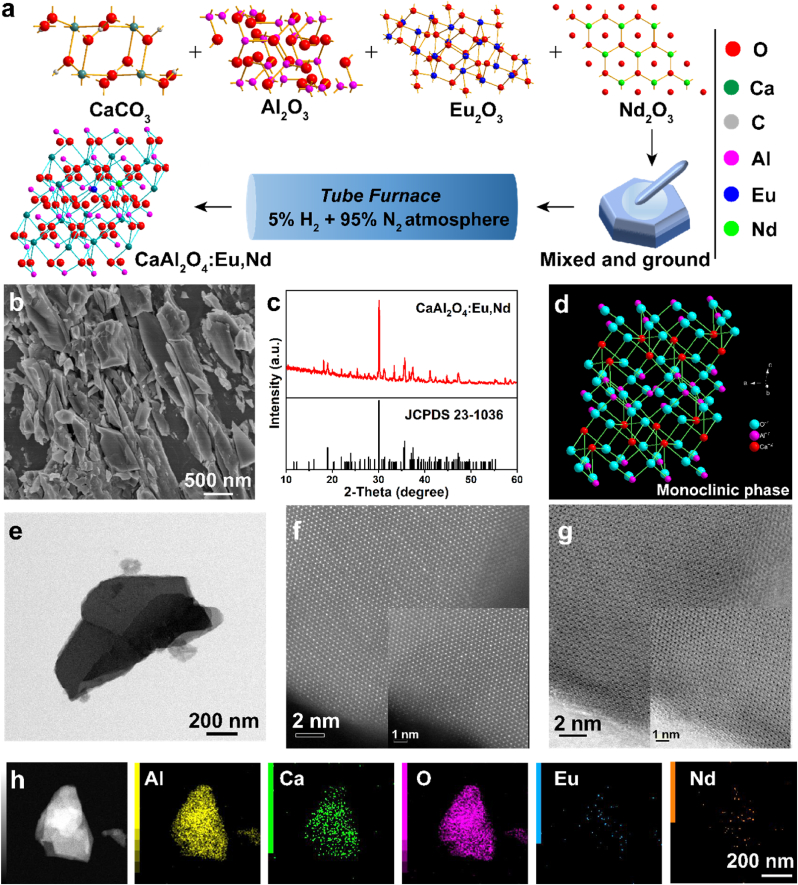
**Morphology and structure characteristics of CAO. (a)** Schematic illustration of the synthestic procedure of CAO. **(b)** SEM image and **(c)** XRD pattern of CAO. **(d)** Crystal structure of CaAl_2_O_4_ host matrix. **(e)** TEM image, **(f)** HAADF-STEM image and **(g)** ABF-STEM image of CAO. The inset images in the lower right corner are the corresponding enlarged HAADF-STEM and ABF-STEM images. **(h)** STEM and the corresponding EDS mapping images of CAO.

The highly crystallized CAO PLM was synthesized via high temperature solid state reaction method under the reduced calcination atmosphere in the presence of CaCO_3_, Al_2_O_3_, Eu_2_O_3_ and Nd_2_O_3_, as depicted in Fig. 1a [33]. Scanning electron microscopy (SEM) image reveals the block-like morphology of products with size ranging from nanometers to several micrometers (Fig. 1b). The characteristic diffraction peaks corresponding to the standard card (JCPDS 23–1036) affirm the formation of calcium aluminate, the effect of rare earth ions (Eu^2+^ and Nd^3+^) doping on the crystal structure is negligible, and the strong diffraction peak intensity indicates the high crystallinity of CAO PLM (Fig. 1c). The crystal structure diagram (ball-and-stick model) intuitively shows the monoclinic phase of CaAl_2_O_4_ host matrix (Fig. 1d). Transmission electron microscopy (TEM) image indicates its sheet-like morphological feature (Fig. 1e). Highly ordered crystal lattice structure and atoms arrangement could be observed through high-angle annular dark field (HAADF) and annular bright field (ABF) STEM images (Fig. 1f and g). The precise elemental analysis process was executed by STEM-energy dispersive spectroscopy (EDS) mapping test (Fig. 1h). The coexistence of Al, Ca, O, Eu, Nd element validates the uniform distribution of corresponding elements in CAO PLM. In addition, the related result is also obtained through EDS mapping affiliated with SEM characterization (Fig. S1). Moreover, the elements and chemical states are further verified through the high-resolution X-ray photoelectron spectroscopy (XPS). The XPS survey scan spectrum confirms the element composition of Al, Ca, O, Eu, Nd. The O 1s high-resolution spectrum can be deconvoluted into two peaks, corresponding to the metal-O bonds (531.2 eV) and oxygen vacancy (530.1 eV), respectively. The Ca 2p_1/2_ and Ca 2p_3/2_ peaks of Ca 2p spin-orbit doublet at 350.8 and 347.3 eV with the splitting energy of 3.5 eV could be captured, demonstrating the existence of Ca^2+^ oxidation state (Fig. S2) [34].

### 3.2 Persistent luminescence property of CAO

**Fig. 2:**
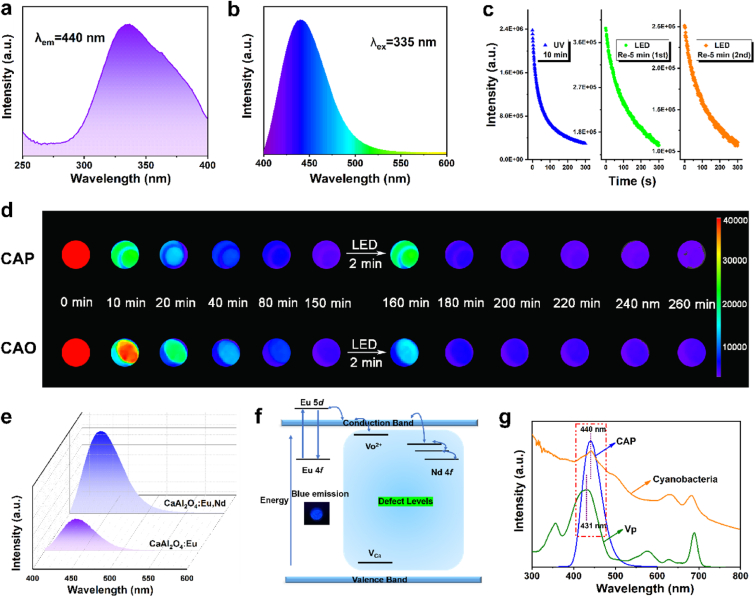
**Persistent luminescence property. (a)** Excitation and **(b)** emission spectra of CAO. **(c)** Afterglow decay curves of CAO powder after repeated excitation with white LED lamp. **(d)** Afterglow luminescence images of CAP and CAO after 10 min irradiation by a 365 nm UV lamp and 2 min re-activation of white LED lamp. **(e)** Emission spectra of CaAl_2_O_4_:Eu,Nd and CaAl_2_O_4_:Eu. **(f)** Scheme of persistent luminescence mechanism of CAO. The inset image refers to the powder of CAO under 365 nm UV irradiation. **(g)** Emission spectrum of CAP and UV–vis absorption spectra of verteporfin (Vp) and cyanobacteria.

To substantiate the persistent luminescence characteristic, the excitation spectrum was initially monitored at 440 nm (Fig. 2a), which presents a single broad excitation band from 300 to 400 nm with the maximum intensity at 335 nm. Subsequently, the emission spectrum of CAO PLM was measured under the excitation of 335 nm (Fig. 2b). The blue broad emission (400–500 nm) arises from the 5d-4f electron transition of Eu^2+^ luminescence center. The “optical battery” feature of CAO PLM can be verified through repeatedly re-activating by white LED lamp (Fig. 2c), this rechargeable blue luminescence of PLM was captured after re-irradiation for 5 min. To meet the biocompatibility requirement, surface functionalization of CAO with polyethylene glycol (PEG) was performed. It has been found that PEGylation induced no significant influence on the afterglow intensity and time compared to pristine CAO PLM (Fig. 2d). It has been well demonstrated that the decay degree of luminescence intensity depends on the atomic state of Eu element, which is less affected by the host matrix. In line with this notion, the structural deformation phenomenon should be avoided. Herein, Eu, Nd co-doped CaAl_2_O_4_ host matrix could meet the above requirement because of the closed radius between Ca^2+^ (1.00 Å) and rare earth ions (Eu^2+^ (1.17 Å) and Nd^3+^ (0.98 Å)). In addition, the fluorescence intensity comparison of Eu-doped CaAl_2_O_4_ and Eu, Nd co-doped CaAl_2_O_4_ exhibited the enhanced positive effect of Nd (Fig. 2e), resulting from the provision of additional electrons from Nd [35]. However, the rapidly decayed afterglow intensity reduces the photodynamic efficiency inevitably. Therefore, it is expected to reduce the fluorescence decay rate by means of ion doping and particle-size control.

To gain deeper insights into the afterglow property, the persistent luminescence mechanism of CAO on the basis of crystal structure and rare earth doping was proposed (Fig. 2f). Firstly, UV irradiation induces the electrons transition process from Eu 4f to 5d energy levels accompanied by the generation of holes. Normally, the returned 5d electrons would cause broad blue light emission. Herein, to meet the persistent luminescence requirement, Eu 5d levels are above the conduction band minimum, which allows the electrons to be stored before moving back to 4f energy levels. It has been demonstrated that some electrons would transfer to the conduction band (CB) and store in O vacancies (V_O_^2+^) as the shallow electron trap [35]. Moreover, Ca vacancies (V_Ca_) as the hole trap center are beneficial for the formation and stabilization of V_O_^2+^ through the reduction of the Fermi level. In addition, Nd 4f level as the deep electron trap would significantly enhance the luminescence intensity [36]. Then, the blue persistent luminescence could be produced through the electron transition to the 5d levels using CB as a bridge, arising from the thermoluminescence effect (Fig. 2f). As one of the vital parts, the overlap of emission spectrum of CAO PLM and the absorption spectrum of photosensitizer verteporfin (Vp) and cyanobacteria plays the determining role in photonic activation. As shown in Fig. 2g, the absorption peak at 440 nm arising from *Chlorophyll a* confirmed the effective photosynthesis activation characteristics of blue light. The high overlap degree ensures that the blue luminescence of CAO could activate both oxygen production and photodynamic effect-induced ^1^O_2_ generation.

### 3.3 Cyanobacteria bioengineering and characterization

**Fig. 3:**
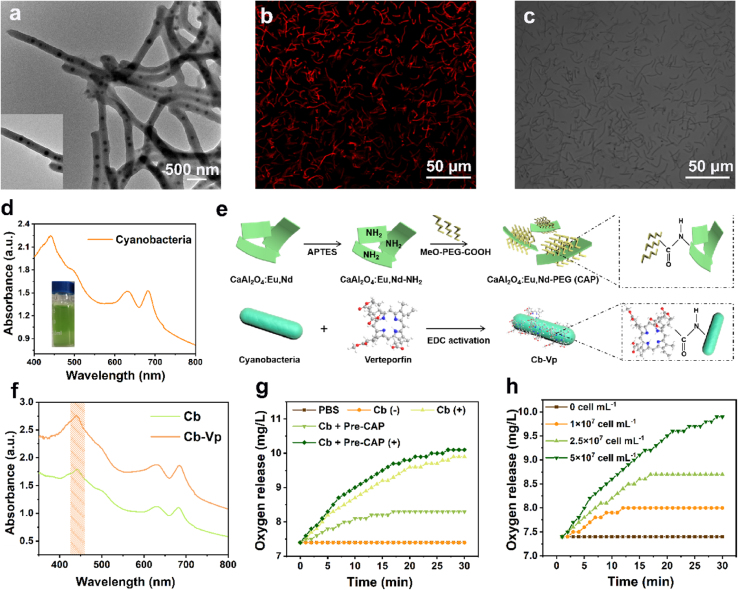
**Cyanobacteria bioengineering O**_**2**_**generation. (a)** TEM image, **(b)** CLSM image and **(c)** microscopic photo of Cb. **(d)** UV–vis absorption spectrum of Cb. The inset image in the lower left corner is the corresponding solution image. **(e)** Scheme of detailed fabrication process of CAP and Cb-Vp. **(f)** The absorption spectra of Cb and Cb-Vp for the calculation of Vp-loading efficiency. **(g)** Dissolved oxygen level variations of solution with different treatments ((−) means in the dark; (+) indicates white LED lamp irradiation). **(h)** Dissolved oxygen level variations of Cb with different cell densities under white LED lamp irradiation.

A typical species of photosynthetic cyanobacteria, namely, *Synechococcus elongatus* PCC 7942, was exploited in this work. Cyanobacteria (abbreviated as Cb) exhibits rod morphology with uniform diameters at the micron level (Fig. 3a). The red autofluorescence property originating from fluorescence phycocyanin and morphological feature of Cb were confirmed by confocal laser scanning microscope (CLSM) (Fig. 3b and c). The absorption spectra of Cb related to oxygen evolution was measured in Fig. 3d. The broad absorption with several strong peaks at 440, 630 and 681 nm can be captured because of the existence of *Chlorophyll a* [37], and Cb cultured in BG11 medium shows green color (inset figure). Moreover, the absorption intensity is positively correlated with the concentration of Cb (Fig. S3). In addition, the rational design and combination of CaAl_2_O_4_:Eu,Nd@PEG (CAP) and cyanobacteria-verteporfin (Cb-Vp) were conducted for alleviating tumor hypoxia and augmenting PDT effect, as illustrated in Fig. 3e. To construct CAP persistent luminescence bio-platform, aminated CAO was initially fabricated through a condensation reaction of (3-aminopropyl)triethoxysilane (APTES) with the hydroxyl groups of CAO. Thereafter, they were functionalized with MeO-PEG-COOH by an amidation reaction to ameliorate the biocompatibility. Zeta potential results verified the desirable preparation of CAP (Fig. S4). For Cb-Vp, the –NH_2_ groups of the outer membrane of Cb were covalently linked with the EDC/NHS-activated photosensitizer Vp by means of the typical amide bonding process. CAP PLM and Cb-Vp are combined in a blending manner throughout the evaluation. The absorption spectra of Vp at different concentrations ranging from 0 to 10 μg mL^−1^ and the corresponding standard concentration curve (Fig. S5) have been measured for the calculation of Vp loading efficiency, which is calculated to be 2.99 wt%, as determined by the absorbance difference of Cb and Cb-Vp at 431 nm (Fig. 3f), manifesting the successful loading of photosensitizer. The visible light absorption characteristics make it feasible to investigate the photosynthesis through quantitative determination of dissolved oxygen. As shown in Fig. 3g, no fluctuation effect was found in pure phosphate buffer solution (PBS, pH = 7.4) without the addition of Cb, manifesting the dynamic equilibrium of dissolved oxygen. The dissolved oxygen level difference between Cb without irradiation (abbreviated as: Cb (−)) and Cb irradiated with white LED lamp (abbreviated as: Cb (+)) solidly demonstrated the visible light-induced photosynthetic oxygen production capability. This “on (with irradiation)/off (without irradiation)” switch function of cyanobacteria ensures its controllable oxygenation. Furthermore, since the overlap of broad blue light emission arising from CAP and absorption peak position of Cb, the exogenous irradiation-free photosynthetic effect has been confirmed through the combination of Cb and UV pre-excited CAP (abbreviated as: Pre-CAP). Importantly, more distinct dissolved oxygen productions are visualized in Cb + Pre-CAP with white LED irradiation (abbreviated as: Cb + Pre-CAP (+)) group compared with Cb (+) alone group, demonstrating the positive effect of Pre-CAP on photosynthetic oxygenation (Fig. 3g). In addition, the results of density correlation oxygenation experiment confirmed the Cb concentration-dependent dissolved oxygen production (Fig. 3h).

### 3.4 *In vitro* oxygenation at cellular level

**Fig. 4:**
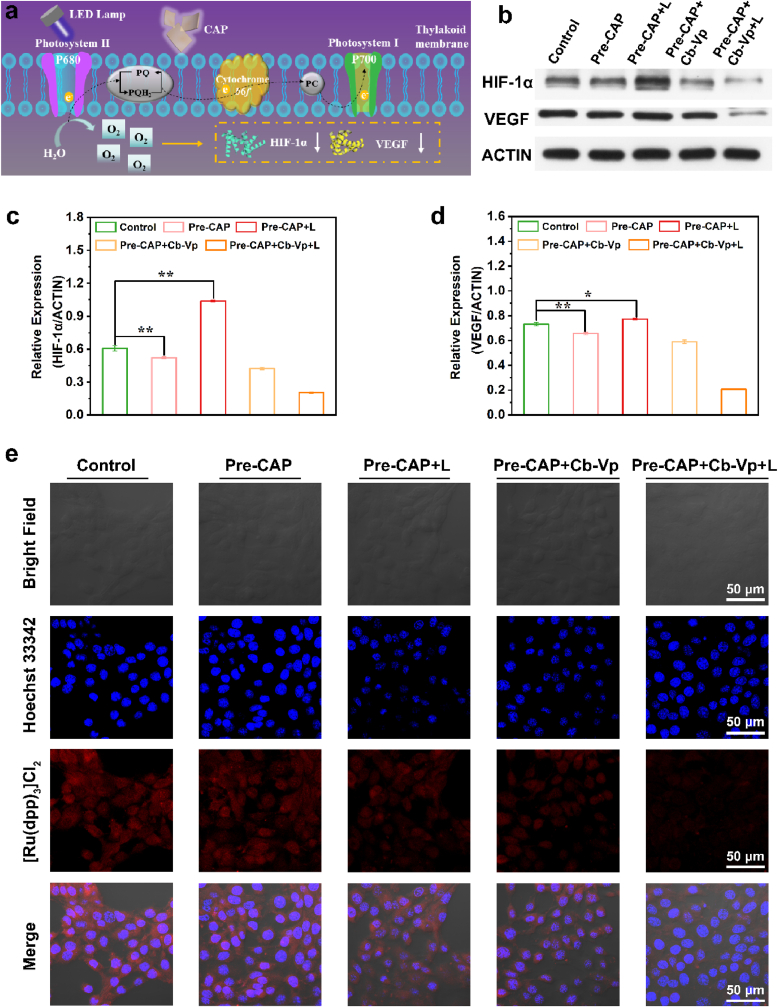
**Hypoxia alleviation *in vitro*. (a)** Schematic diagram of oxygen production by the engineered photosynthesis. **(b)** Western blotting analysis of HIF-1α and VEGF protein levels from 4T1 cancer cells after different treatments. The corresponding quantitative analysis of **(c)** HIF-1α and **(d)** VEGF protein expressions treated with different groups based on western blotting results. **(e)** CLSM images of 4T1 cancer cells exposed to [Ru(dpp)_3_]Cl_2_ probe after different treatments (Pre-CAP [400 μg mL^−1^]; Cb-Vp [Cb:5 × 10^7^ cell mL^−1^, Vp:0.3 μg mL^−1^]; L refers to white LED re-excitation for 2 min).

Based on the aforementioned results involving blue emission-matched photosynthesis of cyanobacteria, we next explored the oxygenation capability of persistent luminescence materials-cyanobacteria self-produced oxygen platform *in vitro* at cellular level. Typically, cyanobacteria has developed a diverse range of electron transport pathways to achieve their photosynthesis and aerobic respiration [38]. As illustrated in Fig. 4a, thylakoid membrane, as the main membrane system, undertakes the critical task of photosynthesis. The whole process begins with photosystem II complexes (P680)-induced water splitting under light irradiation. As one of the products, electrons participate in the synthesis of NADPH through the transfer process from PSII to PSI. Meanwhile and most importantly, oxygen molecules are generated and diffused. Therefore, it is reasonably speculated that the introduction of cyanobacteria would reverse the intracellular hypoxic microenvironment by photosynthetic oxygenation. Correspondingly, the hypoxia-associated protein expressions after different treatments were evaluated to determine the intracellular oxygenation level through the typical western blotting analysis (Fig. 4b). In addition to hypoxia-inducible factor 1 (HIF-1α), we also show solicitude for the expression of vascular endothelial growth factor (VEGF), which is related to angiogenesis, nutrient delivery and tumor growth [5]. Compared with control group and Pre-CAP group, the combination of Pre-CAP and Cb-Vp is competent to trigger the reduction of HIF-1α and VEGF protein expressions, arising from photosynthesis of cyanobacteria induced by blue persistent emission. Furthermore, LED re-excitation process further enhanced the alleviation efficacy of hypoxia, which is the result of the combined effect of the intrinsic white light from LED lamp and the increased blue persistent emission. The corresponding quantitative data analysis affirmed the above results (Fig. 4c and d).

In order to further intuitively confirm the oxygenation effect of self-produced oxygen platform in the cellular microenvironment, the typical oxygen-sensitive probe [(Ru(dpp)_3_)]Cl_2_ with high biocompatibility was exploited to achieve the visualization of cellular O_2_-evolving based on the detection mechanism of oxygen-dependent fluorescence quenching. As displayed in Fig. 4e, the control group exhibits the characteristic bright red fluorescence as a consequence of the inherent cellular hypoxic environment. Meanwhile, distinguished from Pre-CAP group, Pre-CAP + L (LED re-excitation) group and Pre-CAP + Cb-Vp group with a negligible O_2_ level, the fluorescence intensity of [(Ru(dpp)_3_)]Cl_2_-stained 4T1 tumor cells treated with Pre-CAP + Cb-Vp + L group exhibit a sharp decline, confirming the amelioration of hypoxia phenomenon at the cellular level and affirming the vigorous role of white LED re-stimulation (Fig. S6).

### 3.5 *In vitro* therapeutic effect evaluation

**Fig. 5:**
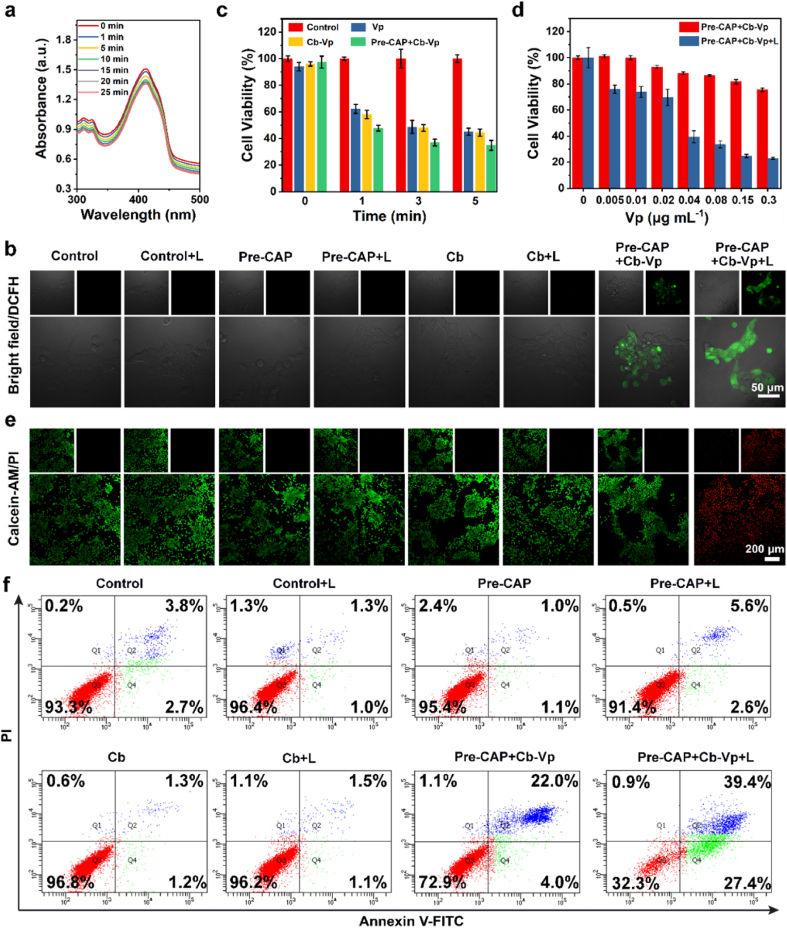
***In vitro* therapeutic effect. (a)**^1^O_2_ production ability. The absorption spectra of DPBF mixed with Pre-CAP and Cb-Vp at different time intervals after the cessation of UV light pre-irradiation (10 min). **(b)** Confocal laser scanning microscope images of 4T1 cancer cells exposed to DCFH-DA after different treatments. **(c)** Relative viabilities of 4T1 cancer cells after being incubated with Vp (0.06 μg mL^−1^), Cb-Vp (Vp: 0.06 μg mL^−1^, Cb: 2 × 10^7^ cell mL^−1^) and Pre-CAP (400 μg mL^−1^) + Cb-Vp (Vp: 0.06 μg mL^−1^, Cb: 2 × 10^7^ cell mL^−1^), then irradiated with white LED light for various durations (1, 3, 5 min). **(d)** Relative viabilities of 4T1 cancer cells incubated with Pre-CAP (400 μg mL^−1^) and Cb-Vp of varied Vp concentrations. L refers to LED re-excitation for 2 min. **(e)** Confocal laser scanning microscope images of Calcein AM (green, live cells)/PI (red, dead cells) co-stained 4T1 cancer cells after different treatments. **(f)** Flow cytometry analysis of 4T1 cancer cells after different treatments through Annexin V-FITC/PI staining. (Pre-CAP [400 μg mL^−1^]; Cb [Cb: 5 × 10^7^ cell mL^−1^]; Cb-Vp [Cb: 5 × 10^7^ cell mL^−1^, Vp: 0.3 μg mL^−1^]; Pre-CAP + L and Pre-CAP + Cb-Vp + L [L refers to LED re-excitation for 2 min]; Cb + L [L refers to LED excitation for 5 min]).

Inspired by the elevated oxygen levels arising from the rational combination of cyanobacteria and CAO PLM, we further verified the therapeutic effect of O_2_-dependent exogenous irradiation-free PDT *in vitro*. Firstly, the changes in absorption intensity of 1,3-diphenylisobenzofuran (DPBF) were monitored to evaluate ^1^O_2_ generation at the solution level. A continual decay of absorption intensity was observed in the mixed solution of Pre-CAP and Cb-Vp, which indicates the formation of ^1^O_2_ (Fig. 5a, Figs. S7–S8). To further validate the augmented generation efficiency of ^1^O_2_ after prolonging the pre-irradiation time, the changes in absorption intensity of DPBF mixed with Pre-CAP (pre-irradiated for 20 min or 30 min) and Cb-Vp were assessed. The reduction ratios of the absorption value are 15.3% and 21.5% with different pre-irradiation time (20 and 30 min), respectively. It is proved that the extension of the pre-irradiation time would induce the enhancement of the afterglow intensity, thereby, promoting the generation of ^1^O_2_ (Fig. S9). Electron spin resonance (ESR) analysis was conducted to determine ^1^O_2_ production, where the typical 1:1:1 triplet ESR signal of ^1^O_2_ could be recorded in the Pre-CAP + Cb-Vp + L group, using 2,2,6,6-tetramethyl-4-piperidone hydrochloride (TEMP) as the trapping agent. Additionally, the extended white light exposure duration increased O_2_ production, in accompany with ESR signal intensity elevation (Fig. S10). In addition, a more intuitive demonstration of ROS generation was conducted by testing the fluorescence intensity of the typical 2′,7′-dichlorofluorescin diacetate (DCFH-DA) probe through CLSM (Fig. 5b). No green fluorescence was observed in 4T1 cancer cells treated with white LED irradiation, Pre-CAP, Pre-CAP + L (LED irradiation), Cb and Cb + L groups. By contrast, the bright green fluorescence of Pre-CAP + Cb-Vp group and Pre-CAP + Cb-Vp + L group provides solid evidences to certify the intracellular ^1^O_2_ production.

Subsequently, we systematically evaluated the intracellular therapeutic efficiency of exogenous irradiation-free photosynthetic bacteria-based PDT platform. Firstly, the relative viabilities of CAP-, Cb- and Vp-treated 4T1 cancer cells with different incubation durations (24 h and 48 h) confirmed their favorable biocompatibility (Figs. S11–S13). Furthermore, light activation time-dependent feature could be captured in the CCK assay results of 4T1 cancer cells (Fig. 5c). Pre-CAP + Cb-Vp group exhibited the minimal cell-killing effect without LED re-activation. However, LED re-activation intensifies the 4T1 cell-killing effect of Pre-CAP + Cb-Vp, causing around 60% cell death ration, which is higher than that of Vp alone or Cb-Vp. Such an elucidation affirmed that the amplified PDT effect originated from the elevated intracellular O_2_ level and continuous output of ^1^O_2_. Meanwhile, restrained cancer-cell proliferation effect would be magnified as the increase in irradiation duration. Besides, the cell damage capability of Pre-CAP + Cb-Vp + L group is positively dependent on the concentration of Cb-Vp (Fig. 5d). Encouraged by the above experimental results, Calcein acetoxymethyl ester (Calcein-AM) and propidium iodide (PI) staining assay was implemented for visual observation (Fig. 5e). The live and dead cells were stained as green and red fluorescence, respectively. As expected, the combination of Pre-CAP and Cb-Vp induced the appearance of part of red fluorescence signal, and the strong red fluorescence could be captured in the Pre-CAP + Cb-Vp + L group, which was consistent with the results of CCK assay. The apoptosis experiments of 4T1 cancer cells were conducted by flow cytometry analysis by the typical fluorescein isothiocyanate (FITC)-labelled Annexin V and propidium iodide (PI) stained method (Fig. 5f, Fig. S14). Similar to control group with high survival rate (93.3%), LED, Pre-CAP, Pre-CAP + L, Cb, Cb + L groups also caused a negligible apoptosis proportion. Comparatively, a certain number of 4T1 cancer cells showed the late apoptosis characteristics (22%) in the Pre-CAP + Cb-Vp group. LED re-activation operation induced a large number of cell apoptosis (early apoptosis: 27.4% and late apoptosis: 39.4%), affirming the amplified PDT therapeutic performance.

### 3.6 *In vivo* therapeutic effect and anticancer mechanism on animal tumor model

**Fig. 6:**
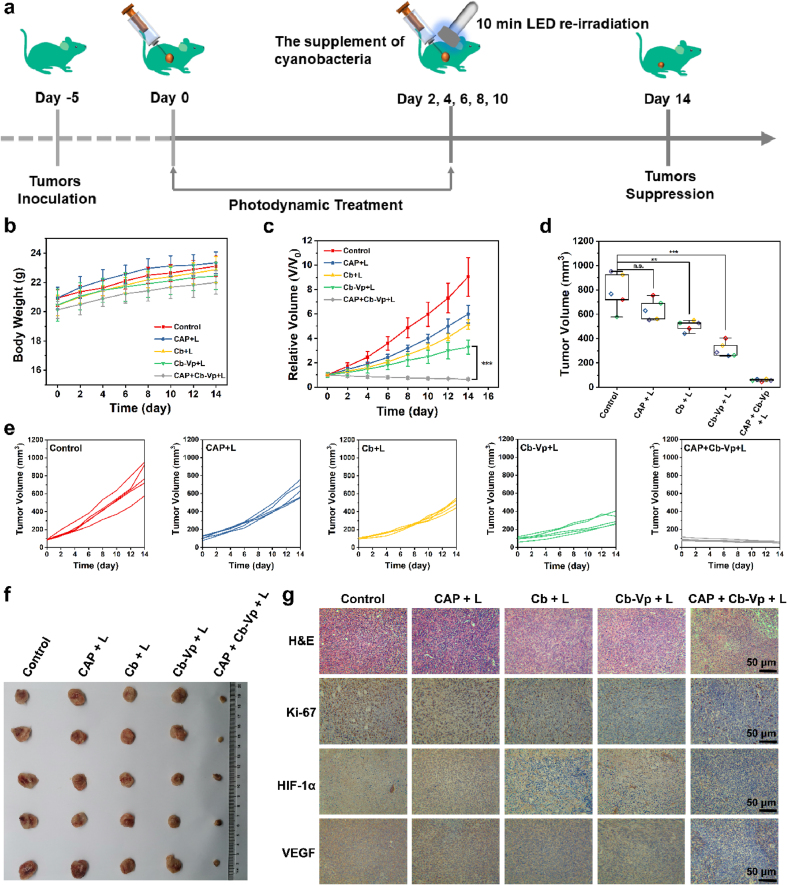
***In vivo* therapeutic effect. (a)** Schematic illustration of exogenous irradiation-free photosynthetic cyanobacteria-based photodynamic tumor therapy (n = 5). **(b)** Time-dependent body weights and **(c)** relative tumor volumes of tumor-bearing mice in different treatment groups during the therapeutic procedure, including control group, CAP + L group, Cb + L group, Cb-Vp + L group and CAP + Cb-Vp + L group. **(d)** Tumor volumes of mice after different treatments at day 14. **(e)** Individual tumor-growth curve of mice after various treatments. **(f)** Representative digital photos of the dissected tumors in various treatment groups. **(g)** H&E, Ki-67, HIF-1α and VEGF staining images of tumor sections after different treatments at day 14.

In view of effective exogenous irradiation-free cyanobacteria-based PDT *in vitro*, the antineoplastic experiments *in vivo* were then performed on 4T1 xenograft tumor-bearing female BALB/c mice models, which were divided into five groups randomly, including control, CAP + L, Cb + L, Cb-Vp + L and CAP + Cb-Vp + L groups (n = 5 per group). In this part, CAP refers to CAP as pre-excited by UV lamp, and L represents white LED lamp irradiation. It is worth noting that light attenuation during tissue penetration process is one of the problems faced by photodynamic tumor therapy. Therefore, the subcutaneous tumor model was herein established to eliminate worries about the penetration depth. The varied treatments were carried out when the tumor size reached ~100 mm^3^. The supplement of cyanobacteria and the re-excitation of LED lamp were performed at day 2, 4, 6, 8, 10 (Fig. 6a). Firstly, the desirable *in vivo* biocompatibility of exogenous irradiation-free PDT system was verified through negligible body-weight fluctuations over a period of 14 days (Fig. 6b). Concurrently, the main organs (heart, liver, spleen, lung, and kidney) of mice treated with different groups were separated for biosafety assessment by hematoxylin and eosin (H&E) staining. No distinct damage or inflammation was observed in histocompatibility assessment (Fig. S15). The tumor volumes were recorded by a digital vernier caliper during the treatment period (Fig. 6c, e). Different from the control group with remarkable 4T1 breast tumor-growth rate, marked tumor-suppression efficiency was monitored in mice treated with Cb-Vp under white LED irradiation, which affirmed the synergistic tumor-killing roles of photosynthetic oxygenation and subsequently ^1^O_2_ production induced by white light irradiation. Meanwhile, CAP PLM exacerbated tumor growth inhibition because of the persistent luminescence property-augmented self-oxygenation PDT without the aid of long-term exogenous irradiation. Subsequently, tumors were collected and weighted after 14 days of treatment, which was quite reconcilable with the results of tumor growth curves (Fig. 6d, f). Moreover, the therapeutic efficacies were quantitatively assessed through the tumor growth inhibition (TGI) rates (Fig. S16). Compared with the relative TGI rates derived from CAP + L group (33.8%) and Cb + L group (44.3%), Cb-Vp + L group increased the TGI rate to 63.7% at the end of day 14. Notably, CAP + Cb-Vp + L group with the distinct inhibition rate (92.9%) affirmed the antineoplastic effect of exogenous irradiation-free PDT nanoplatform.

The typical H&E staining approach was applied to evaluate 4T1 cancer-cells destruction degree of tumor slices, where the obvious histological damages emerged in CAP + Cb-Vp + L group (Fig. 6g). Ki-67 immunohistochemistry and transferase dUTP nick end labelling (TUNEL) staining assay (Fig. S17) also validated the poor tumor cell proliferation ability and a higher degree of apoptotic cells in the Cb-Vp + L group and CAP + Cb-Vp + L group, respectively. In addition, given that hypoxia is a hallmark of tumors and the oxygenation capability of the developed CAP + Cb-Vp system, immunohistochemical assays of HIF-1α and VEGF of tumor sections were carried out, as shown in Fig. 6g. Distinguish from control group and CAP + L group, the employment of cyanobacteria induced the significantly decreased HIF-1α and VEGF expression levels, confirming the relief of hypoxia in the tumor area. All these *in vivo* antitumor assessment results are supportive of the outstanding antineoplastic efficacy of the engineered exogenous irradiation-free photosynthetic bacteria-based PDT platform.

## 4 Conclusions

In summary, we have successfully designed and engineered an exogenous irradiation-free platform for oxygen-boosted PDT through the rational combination of photosensitizer, photosynthetic cyanobacteria and blue-emitting PLM. PLM with the intrinsic energy storage characteristic simultaneously activates cyanobacterial cells for photosynthetic oxygenation and photosensitizer for photodynamic tumor therapy, which maintained the tumor microenvironment at an oxygen-rich level (hypoxia alleviation) and induced ^1^O_2_ generation (augmented photodynamic effect) without requiring the conventional long-term exogenous light excitation, solving the critical issue of low tissue-penetrating depth of exogenous light activation and avoiding the potential side effects of prolonged light exposure. Both *in vitro* and *in vivo* results confirmed the blue persistent luminescence-stimulated O_2_ generation capacity and subsequently improved PDT therapeutic outcomes. This exogenous irradiation-free strategy solves the dual problems of tumor hypoxia and exogenous light source for photodynamic tumor therapy, providing a specific paradigm of microbial-based nanotherapy with the assistance of rational design, engineering and integration of persistent luminescence phosphors as the desirable light irradiation source.

## CRediT authorship contribution statement

**Meiqi Chang:** Conceptualization, Methodology, Investigation, Writing – original draft. **Wei Feng:** Investigation. **Li Ding:** Investigation. **Hongguang Zhang:** Visualization. **Caihong Dong:** Conceptualization, Supervision, Visualization, Writing – review & editing. **Yu Chen:** Conceptualization, Supervision, Writing – review & editing, Funding acquisition. **Jianlin Shi:** Supervision, Writing – review & editing, Funding acquisition.

## Declaration of competing interest

The authors declare that they have no known competing financial interests or personal relationships that could have appeared to influence the work reported in this paper.
