# Supplementary material for: Persistent luminescence phosphor as in-vivo light source for tumoral cyanobacterial photosynthetic oxygenation and photodynamic therapy
Source: Bioact Mater. 2021 Sep 4;10:131–44. doi: 10.1016/j.bioactmat.2021.08.030 (PMC8637009; doi:10.1016/j.bioactmat.2021.08.030)
Supplement: Multimedia component 1 [file mmc1.docx]

**Supporting Information**

Persistent Luminescence Phosphor as *In-vivo* Light Source for Tumoral Cyanobacterial Photosynthetic Oxygenation and Photodynamic Therapy

Meiqi Chang^1^, Wei Feng^2^, Li Ding^1^, Hongguang Zhang^3^, Caihong Dong^4,^*, Yu Chen^1,2,^* and Jianlin Shi^1^

^1^State Key Laboratory of High Performance Ceramics and Superfine Microstructure, Shanghai Institute of Ceramics, Chinese Academy of Sciences, Shanghai 200050, P. R. China.

^2^Materdicine Lab, School of Life Sciences, Shanghai University, Shanghai 200444, P. R. China

Email: chenyuedu@shu.edu.cn.

^3^College of Pharmacy, Qiqihar Medical University, Qiqihar 161006, P. R. China.

^4^Department of Ultrasound, Zhongshan Hospital, Fudan University, and Shanghai Institute of Medical Imaging, Shanghai, 200032, P. R. China.

Email: dong.caihong@zs-hospital.sh.cn.

**Supplementary figures**


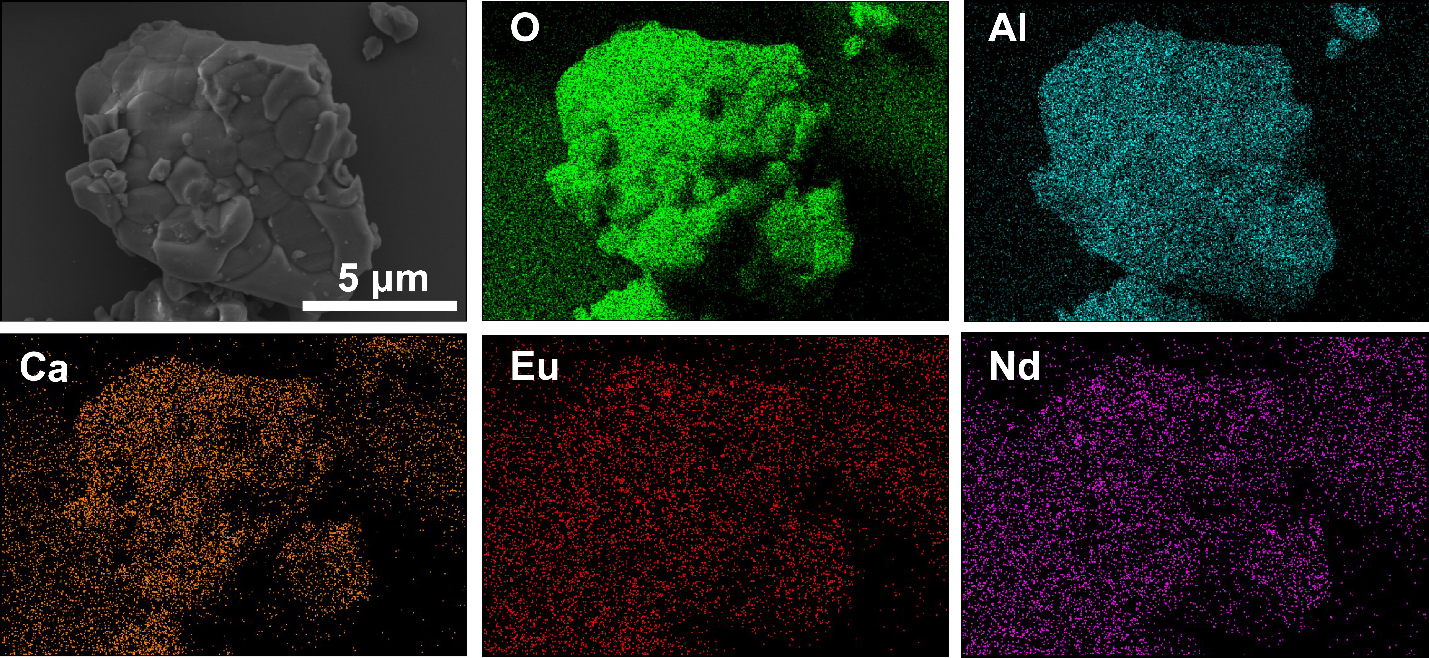


**Fig. S1** SEM image of CaAl_2_O_4_: Eu, Nd and corresponding EDS mapping of O, Al, Ca, Eu and Nd.


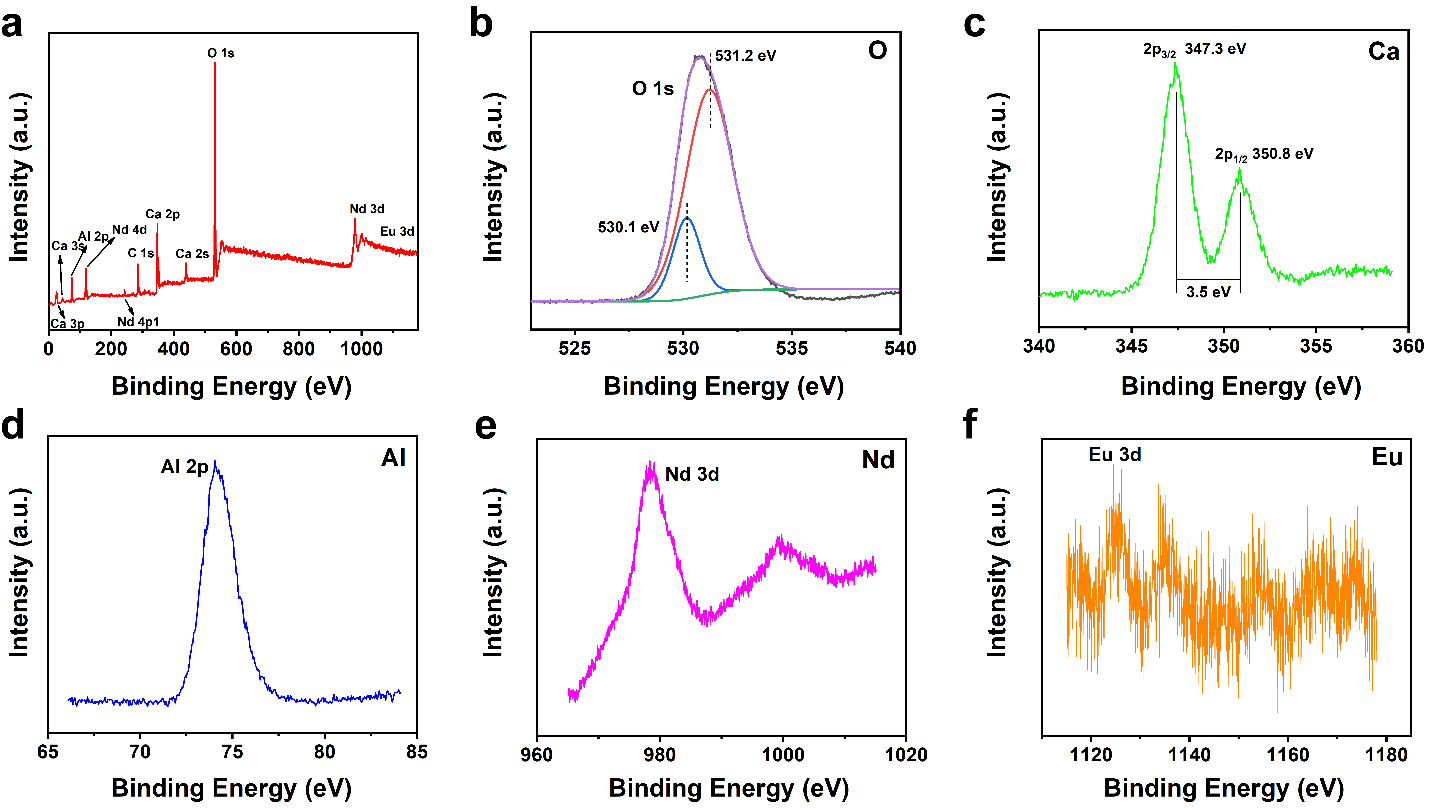


**Fig. S2** XPS spectra of (a) wide-scan, (b) O 1s, (c) Ca 2p, (d) Al 2p, (e) Nd 3d and (f) Eu 3d of CaAl_2_O_4_: Eu, Nd.


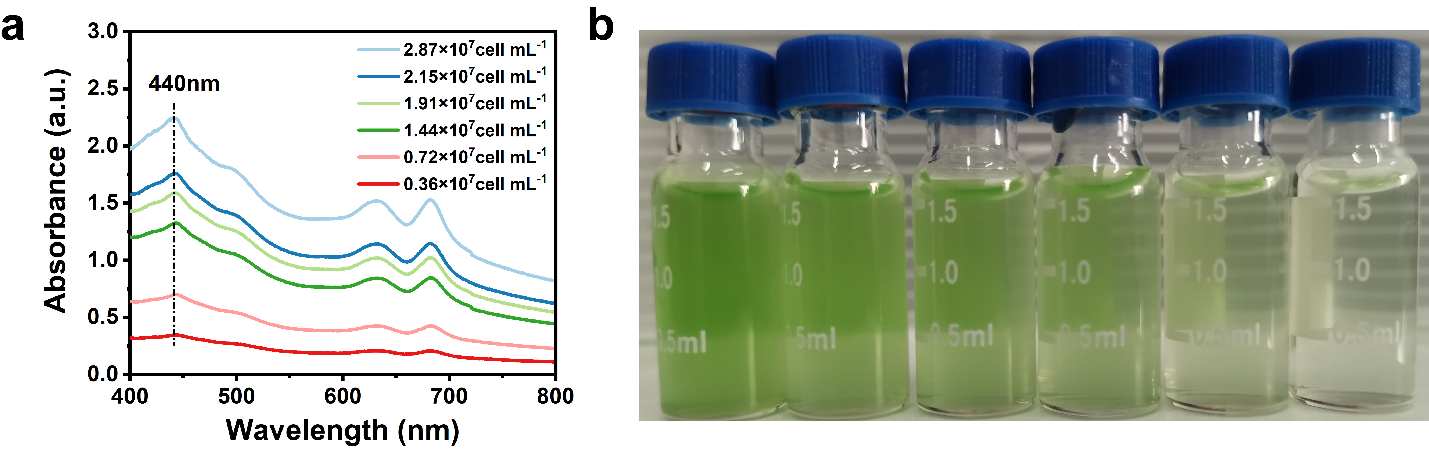


**Fig. S3** (a) Absorption spectra of cyanobacteria at different density. (b) Corresponding digital picture of cyanobacteria solution.





**Fig. S4** Zeta potential distribution in deionized water for CAO, CAO-OH, CAO-NH_2_ and CAP.


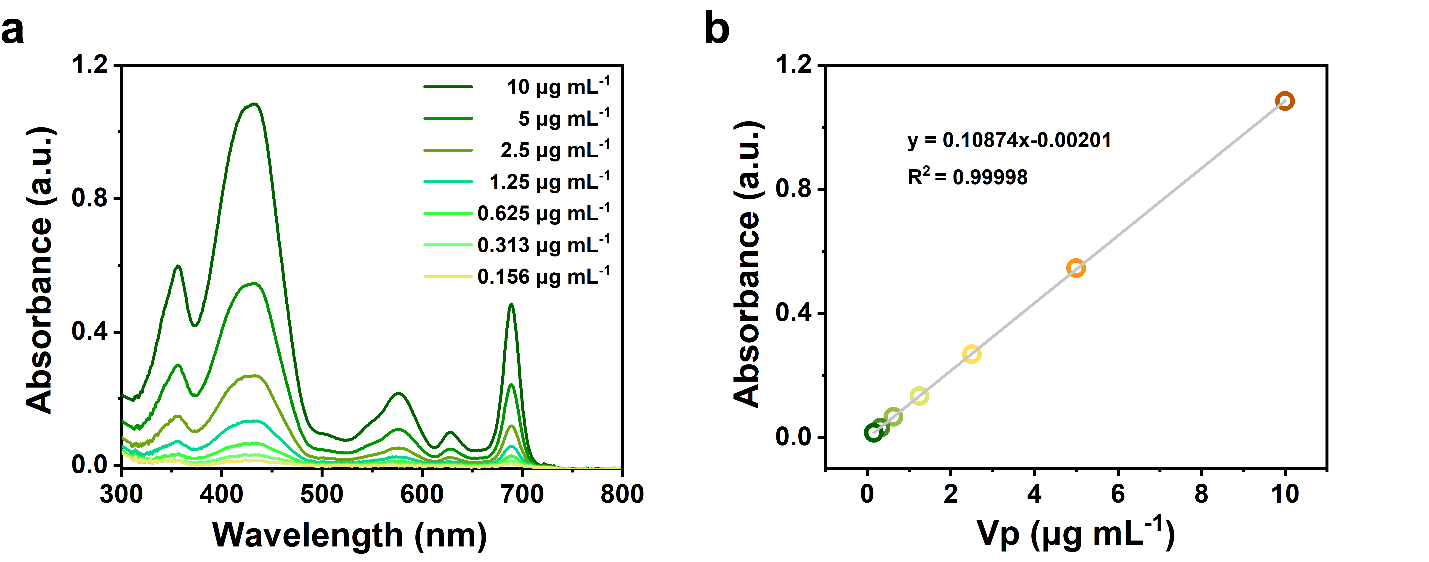


**Fig. S5** (a) The absorption spectra of Vp at different concentrations ranging from 0 to 10 μg mL^-1^. (b) The standard concentration curve obtained by fitting the concentration-dependent absorbance data of Vp at 431 nm.





**Fig. S6** Mean fluorescence intensity of 4T1 cancer cells exposed to [Ru(dpp)_3_]Cl_2_ probe after different treatments determined by Image J software (Pre-CAP [400 µg mL^-1^]; Cb-Vp [Cb:5×10^7^ cell mL^-1^, Vp:0.3 µg mL^-1^]; L refers to white LED re-excitation for 2 min).





**Fig. S7** Corresponding absorbance values of DPBF mixed with Pre-CAP and Cb-Vp at different time intervals after the cessation of the 365 nm UV light pre-irradiation (10 min).


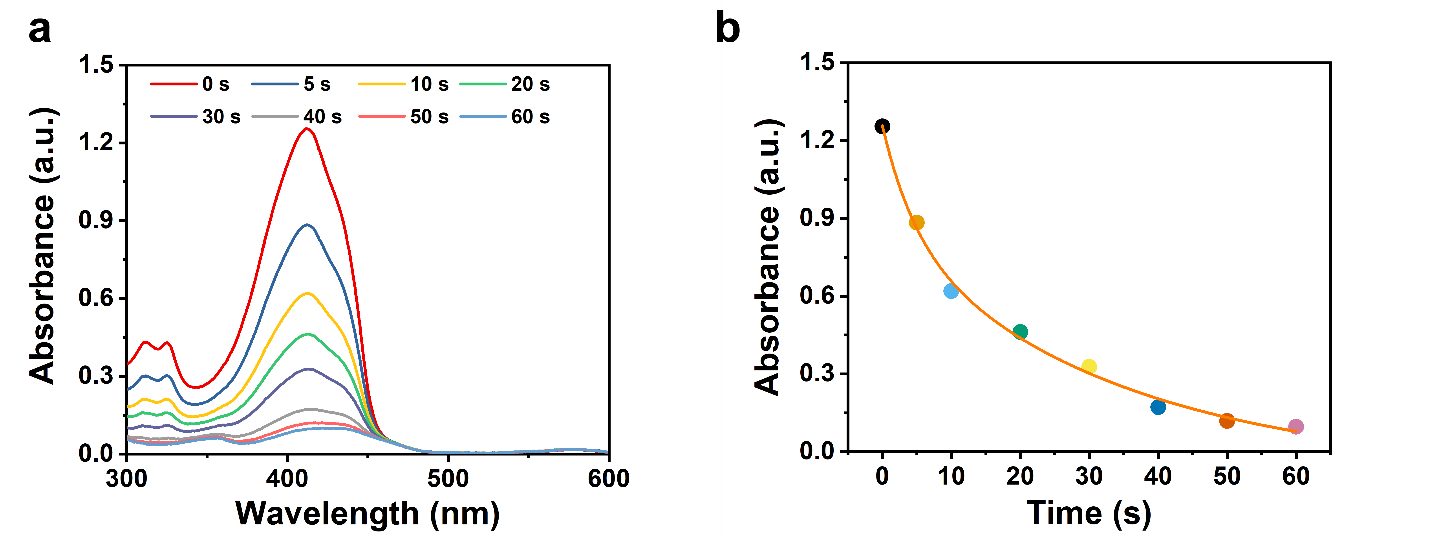


**Fig. S8** (a) The absorption spectra of DPBF mixed with Pre-CAP and Cb-Vp at different time intervals upon irradiation with a white LED lamp. (b) The corresponding absorbance values at different time intervals.


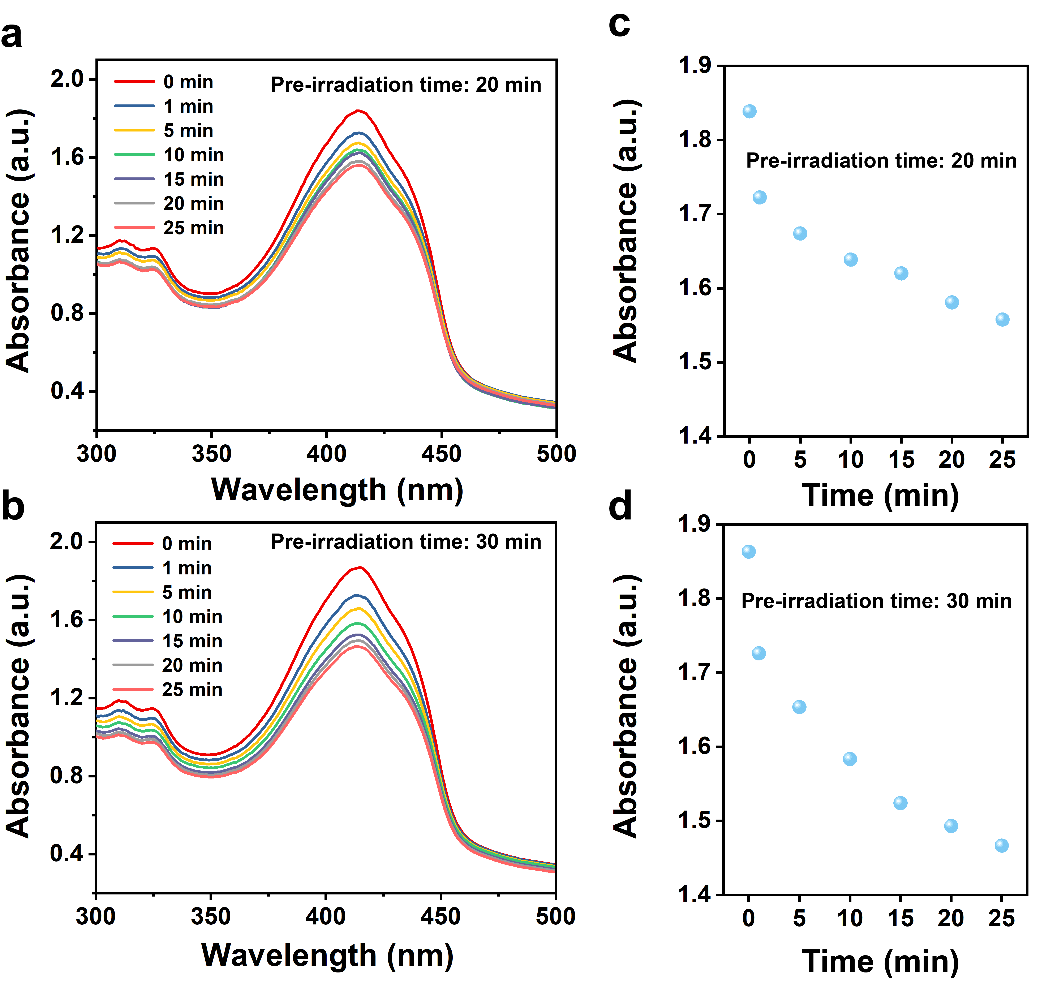


**Fig. S9** The absorption spectra (a and b) and the corresponding absorbance values (Wavelength: 413 nm) of DPBF (c and d) mixed with Pre-CAP and Cb-Vp at different time intervals after the cessation of the 365 nm UV light pre-irradiation (20 min and 30 min).





**Fig. S10** ESR spectra of the mixture of CAP and Cb-Vp with different LED irradiation durations in the presence of TEMP.

**

**

**Fig. S11** Relative viabilities of 4T1 cancer cells after being incubated with CAP with varied concentrations for 24 h and 48 h.





**Fig. S12** Relative viabilities of 4T1 cancer cells after being incubated with cyanobacteria with varied concentrations for 24 h and 48 h.





**Fig. S13** Relative viabilities of 4T1 cancer cells after being incubated with Vp with varied concentrations for 24 h and 48 h.


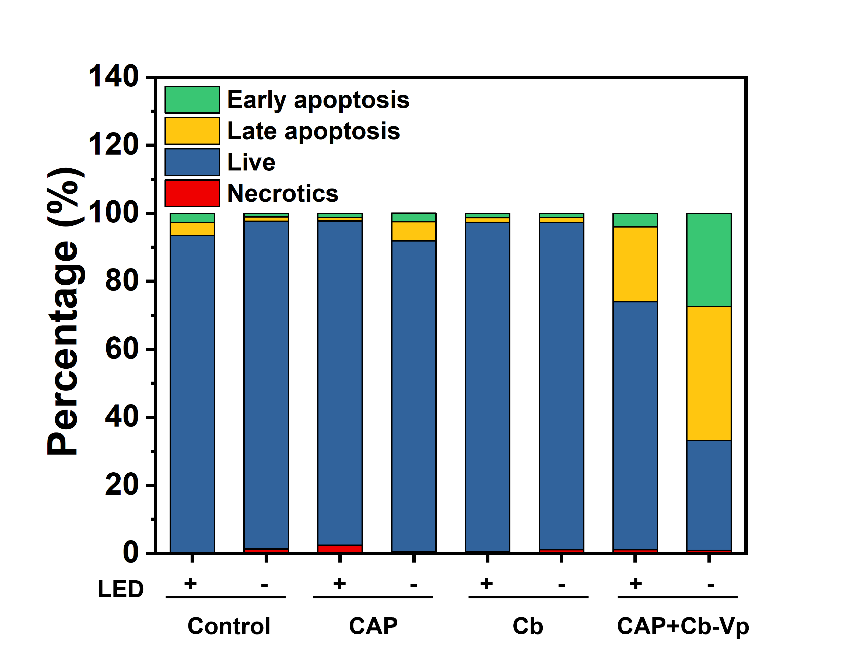


**Fig. S14** Flow cytometry quantitative analysis of 4T1 cancer cells after different treatments through Annexin V-FITC/PI staining.


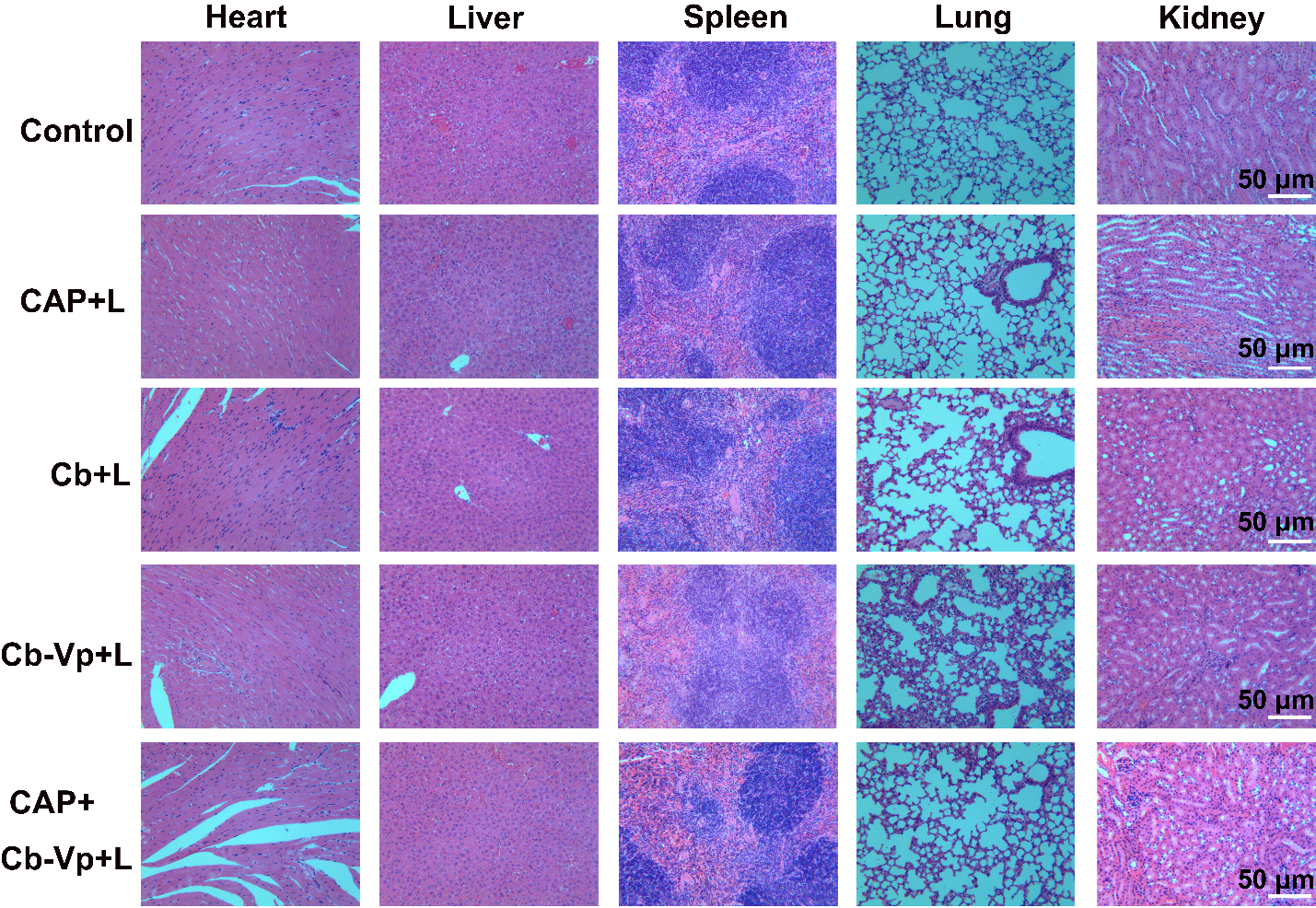


**Fig. S15** H&E staining images of the major organs (heart, liver, spleen, lung, and kidney) from the different treatment groups.





**Fig. S16** The relative tumor inhibition rates of treatment groups (CAP + L, Cb + L, Cb-Vp + L and CAP + Cb-Vp + L) as compared to control group.


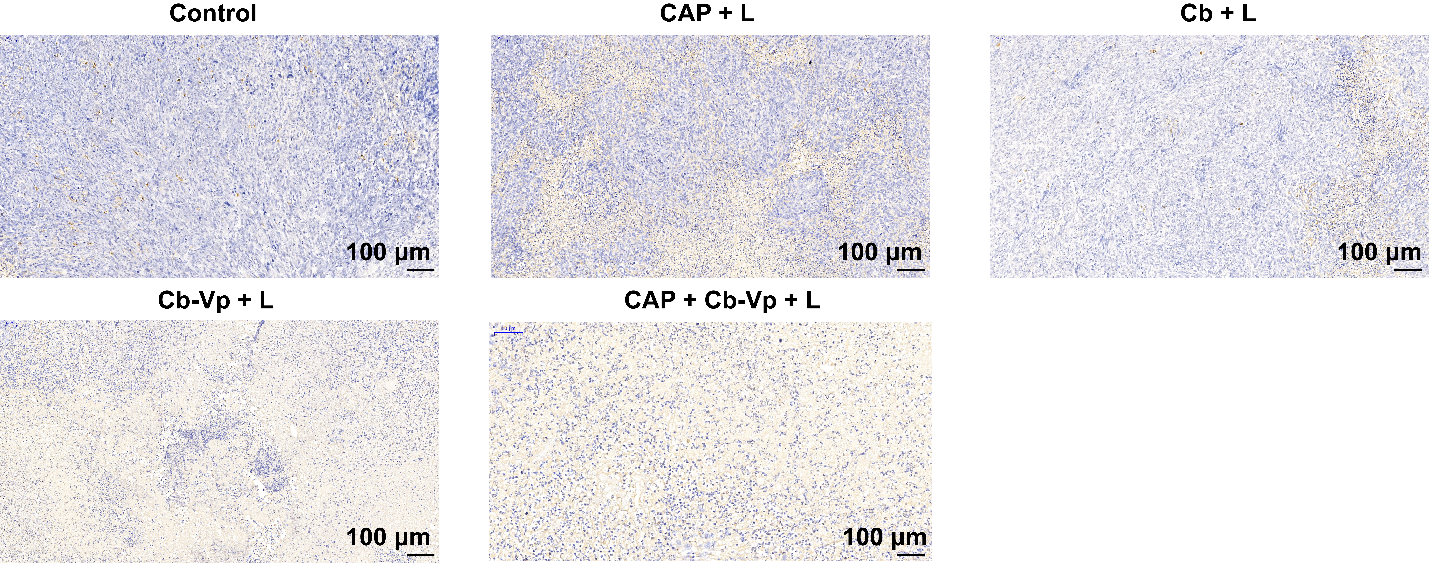


**Fig. S17.** Representative tumor sections stained with TUNEL assay after different treatments at day 14.
